# Structure-based docking, pharmacokinetic evaluation, and molecular dynamics-guided evaluation of traditional formulation against SARS-CoV-2 spike protein receptor bind domain and ACE2 receptor complex

**DOI:** 10.1007/s11696-021-01917-z

**Published:** 2021-10-18

**Authors:** B. Harish kumar, Suman Manandhar, Chetan H. Mehta, Usha Y. Nayak, K. Sreedhara Ranganath Pai

**Affiliations:** 1 Department of Pharmacology, Manipal College of Pharmaceutical Sciences, Manipal Academy of Higher Education, Manipal, Karnataka 576104 India; 2 Department of Pharmaceutics, Manipal College of Pharmaceutical Sciences, Manipal Academy of Higher Education, Manipal, Karnataka 576104 India

**Keywords:** SARS-CoV-2, Kabasura kudineer, Shwas kuthar rasa, Talisadi churna, Computational docking study, Molecular dynamics

## Abstract

There is an urgent need for reliable cure and preventive measures in this hour of the outbreak of SARS-CoV-2. Siddha- and Ayurvedic-based classical formulations have antiviral properties and great potential therapeutic choice in this pandemic situation. In the current study, *in silico*-based analysis for the binding potential of phytoconstituents from the classical formulations suggested by the Ministry of Ayush (Kabasura Kudineer, Shwas Kuthar Rasa with Kantakari and pippali churna, Talisadi churna) to the interface domain of the SARS-CoV-2 receptor-binding domain and angiotensin-converting enzyme 2 was performed. Maestro software from Schrodinger and tools like Glide Docking, induced fit docking, MM-GBSA, molecular dynamics (MD) simulation, and thermal MM-GBSA was used to analyze the binding of protein PDB ID:6VW1 and the selected 133 ligands in comparison with drug molecules like favipiravir and ribavirin. QikProp-based ADMET evaluation of all the phytoconstituents found them nontoxic and with drug-like properties. Selection of top ten ligands was made based on docking score for further MM-GBSA analysis. After performing IFD of top five molecules iso-chlorogenic acid, taxiphyllin, vasicine, catechin and caffeic acid, MD simulation and thermal MM-GBSA were done. Iso-chlorogenic acid had formed more stable interaction with key residue among all phytoconstituents. Computational-based study has highlighted the potential of the many constituents of traditional medicine to interact with the SARS-CoV-2 RBD and ACE2, which might stop the viral entry into the cell. However, *in vivo* experiments and clinical trials are necessary for supporting this claim.

**Supplementary Information:**

The online version contains supplementary material available at 10.1007/s11696-021-01917-z.

## Introduction

The emergence of the global pandemic caused by the novel coronavirus (COVID-19) with the epicenter in Wuhan, China, has claimed many lives and affected the world's population. Till September 26, 2021, more than 232,349,581 people have contracted corona infection, and mortality of 4,758,617 people has been registered in the world due to SARS-CoV-2 (2021a). Coronaviruses have already been a cause for two pandemics, i.e., Middle East respiratory syndrome (MERS) and severe acute respiratory syndrome (SARS), recently in the past two decades. SARS-CoV-2 member of β-coronavirus shares 96.2% genome sequence similarity with bat coronavirus (CoV RaTG13), 79.5% with SARS-CoV, indicating that SARS-CoV-2 might have been transmitted from bats (Guo et al. 2020). Angiotensin-converting enzyme 2 (ACE 2), serving as a cell receptor for entry of SARS-CoV, was also a receptor for S glycoprotein present in the surface of SARS-CoV-2 (Zhou et al. 2020). CryoEM-based analysis of SARS-CoV-2 spike structure has shown the binding affinity of S-protein and ACE2 for SARS-CoV-2 is 10–20 times higher than SARS-CoV. The spike protein receptor-binding domain of SARS-CoV-2 possesses only 40% of sequence identity with other SARS-CoVs (Cascella et al. 2020). Therefore, blocking the interaction between spike protein of ACE2 receptor has been one of the major mechanisms through which enter of the virus into the cell can be prevented (Huang et al. 2020).

Social distancing, quarantine of the infected, and symptomatic treatment for the infected are currently being followed to manage the disease. No allopathic antiviral medications are available to treat this novel disease. Several attempts for vaccine development, repurposing of the known drugs have been tried out. Chloroquine (Singh and Vijayan 2020), a combination of hydroxychloroquine and azithromycin (Gautret et al. 2020), antiviral drugs like ritonavir, lopinavir have been used for treatment and found to be effective in reducing viral load and recovery. However, WHO has discontinued the trials with hydroxychloroquine and lopinavir/ritonavir on July 4, 2020, based on the observation that there was no reduction in the mortality rate of COVID-19 patients after the recommendation from the Solidarity Trial’s International Steering Committee (Margaret Harris; Daniela Bagozzi 2020).

India reported its first case on January 30, 2020. There were 10.8 million cases and 154 thousand death reports till Jan 2021(2021b). Owing to the vast population and fewer doctors per 1000 people and hospital beds in India. The impact of SARS-CoV-2 on the Indian health system is enormous and challenging (Puthiyedath et al. 2020). Therefore, in this hour of need, the optimal use of traditional knowledge and AYUSH can ensure the fulfillment of scarcity in the country's health system. WHO has suggested accessing the health care system's infection prevention and control capacity, including traditional practice, healers, and pharmacy in the guidelines to support country preparedness and response (2020a). The majority of SARS-CoV-2 infected people (80%) have mild symptoms (fever, fatigue, shortness of breath, loss of smell, and taste) and can be easily managed with primary medical care. Around 15% require urgent medical care with secondary health care service, and 5% with acute respiratory distress syndrome (ARDS) require critical care in the intensive care unit (Rastogi et al. 2020).

Due to the urgent need for the treatment of the COVID, the molecules need to be selected, which can be useful and at the same time, should be safer. In this case, *in silico* modeling helps select the potential molecule efficiently within less time. Nowadays, all researchers use computational tools to reduce the workload and fasten the drug discovery process. Using the *in silico* technique, rapid analysis of a vast database using high throughput virtual screening can be done with the least time possible. In addition to this, researchers can also study the effect of the drug on the structure of the protein.

Ayurveda, Siddha, and other traditional systems, widely being practiced since ancient times, have an immense role in strengthening the human mind and body. Incorporation of allopathic medication in combination with Rasayana-based therapeutics can provide both prophylactic as well as therapeutic relief for SARS-CoV-2. The Government of India, Ministry of Ayush, has provided Guidelines for the registered practitioners from Ayurveda, Yoga, Unani, Siddha, Homeopathy, and naturopathy system to manage SARS-CoV-2 (2020b, 2020c) The Antiviral Siddha formulation, Kabasura Kudineer, has been suggested as a preventive medication for Siddha practitioners (Kiran et al. 2020). It has been described in ‘Citta Vaittiyattirattu,’ Siddha manuscript for treating phlegmatic fever (Aiyacuram), and Swine flu (Kaba Suram (Swine Flu), n.d.).

Similarly, Ayush guidelines for managing ARDS-like symptoms in SARS-CoV-2 condition for Ayurveda practitioners include Shwas Kuthar Rasa with Kantakari and Pippali churna, Talisadi churna. Talisadi churna, a classical formulation from Astanga Hridaya-Rajayakshma Chikitsa, is advised in acute and allergic bronchitis and exacerbated asthma attacks (Patra et al. 2011). Shwas kuthar rasa, a formulation based on Rasa aushadhis, has been prescribed for moderate to severe symptoms of SARS-CoV-2. In this current study, we have chosen the classical formulations from the guidelines provided by Ayush and evaluated the potential of the active constituents for the inhibition of the ACE2 receptor so that the entry of the SARS-CoV-2 virus can be prevented.

## Materials and methods

All the computational calculations were performed using Schrodinger Maestro Suit using different modules such as LigPrep, SiteMap, Protein Preparation Wizard, Grid Generation, Glide Docking, MM-GBSA, induced fit docking, and Desmond molecular dynamic simulation.

### Ligand preparation

The structure of chief constituents of the plants used in the classical formulations, namely Kabasura Kudineer (Kiran et al. 2020), Shwas Kuthar Rasa with Kantakari (Janadri et al. 2015) and Pippali churna, Talisadi churna (Tekuri et al. 2019), was downloaded from PubChem. These formulations were mentioned in the guidelines provided by Ayush for Ayurvedic and Siddha practitioners. A total of 133 selected ligands were prepared using the LigPrep tool to get the geometry optimized structures at pH 7.0 ± 2.0 with the chirality of the ligand determined by its 3D structure (Schrödinger 2018–3, LLC, New York).

### Protein preparation, sitemap, and grid generation

**Fig. 1:**
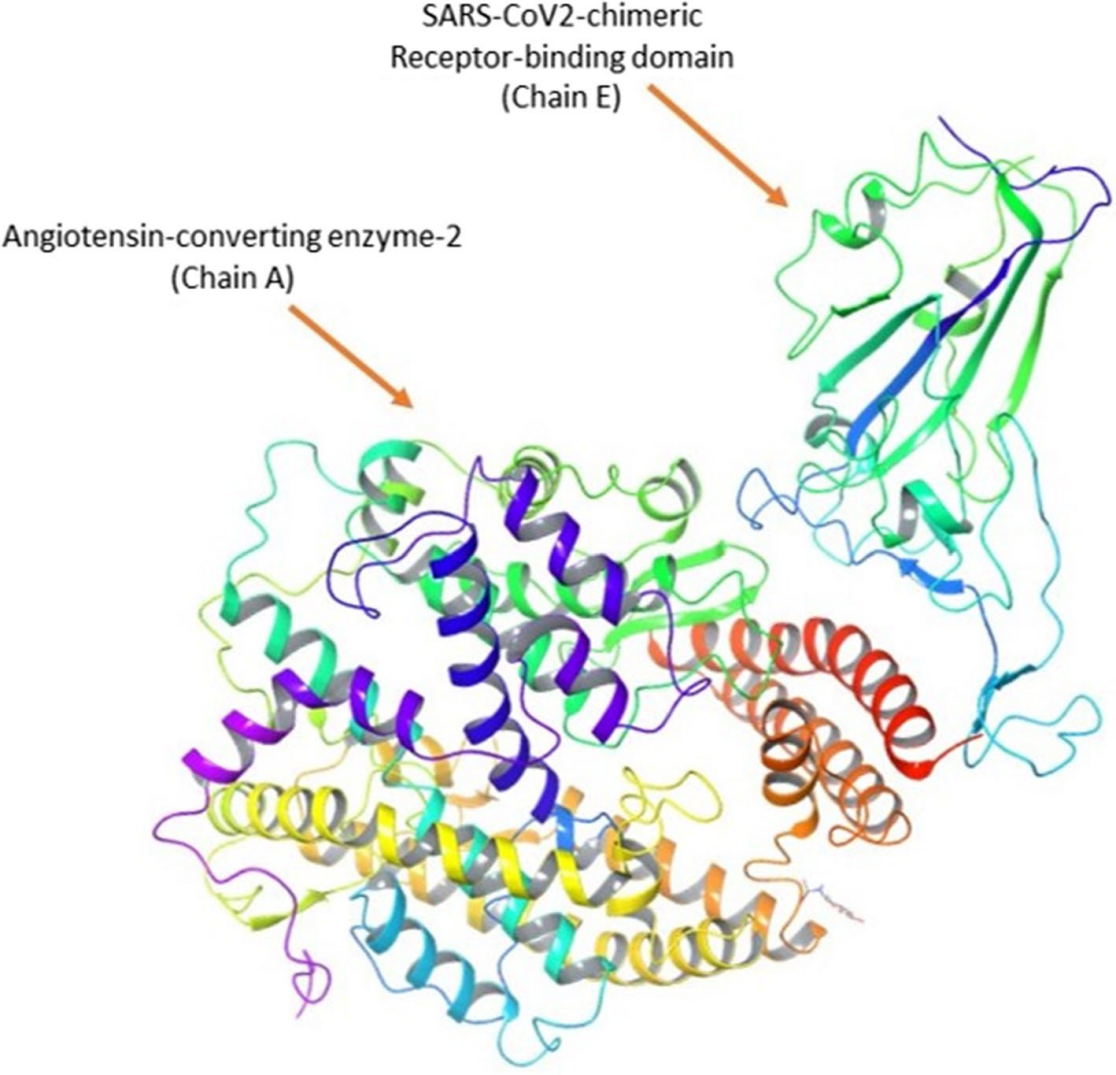
Structure of protein complex (PDB ID 6VW1) containing SARs-Cov-2 chimeric receptor-binding domain (chain E) and angiotensin-converting enzyme 2 (Chain A)

The protein crystal structure of the SARS-CoV-2 Chimeric Receptor-binding domain and Angiotensin-converting enzyme 2 (PDB ID: 6VW1) (Shang et al. 2020) required for performing the computational study was collected from the Protein Data Bank). The Schrodinger module, namely Protein Preparation Wizard, was used for processing the protein structure where it performed in three steps, such as import and process, review, and modify, followed by refinement. In the first step, the protein structure was processed by adding the hydrogen and missing side chains and assigning the bond orders. In the second step, the protein was reviewed for the presence of required side chains, only side chains A and E were selected, and the protein was modified by deleting other side chains. In the last step or third step, H-bonds were assigned and optimized, followed by the removal of water molecules which beyond 3.0 Å. Finally, the restrained minimization was performed for the modified protein by using the OPLS3e force field. As the PDB structure contained many chains, only Chain E, which is SARS-CoV-2 chimeric RBD and Chain A ACE2 receptor, was retained as shown in Fig. 1, and other chains were deleted. The selected protein was without the bound ligand structure; hence SiteMap was performed, which helps identify the probable binding site for the ligand (Halgren 2009). The binding site, which showed the highest site score and was present in the interphase of Chain A and E, was selected for further grid generation using the Glide—Receptor Grid Generation module. The remaining parameters were used by default (Halgren 2007; Madhavi Sastry et al. 2013).

### Molecular docking and binding free energy calculation

The molecular docking was performed by docking the prepared ligands on the selected site of the protein using the Glide module. The receptor grid file and the ligands to be docked from the workspace were selected, and the calculation was performed using extra precision docking. The results were analyzed based on the docking score and molecular interaction formed between the ligand and the protein molecule. The best ligands molecules were selected and subjected to the MM-GBSA and induced fit docking (Halgren et al. 2004; Friesner et al. 2006). Selected ligand and protein structures were considered for performing the MM-GBSA calculations. All the calculations were performed by using VSGB (variable surface generalized born) as the solvent model and OPLS3e as the force field. This calculation helps in calculating the relative binding affinity of the ligands toward the selected protein.

### Induced fit docking (IFD)—extra precision

The extra precision induced fit docking was performed by docking the selected ligands on the rigid protein using the induced fit docking module. While performing the IFD, the receptor and ligand van der Waals scaling was maintained to 0.50 and generated the maximum of 20 poses of the protein with ligands. The calculations were performed by considering the standard precision protocol. The best pose of the ligand–protein complex was selected for performing the MD simulation (Sherman et al. 2006b, 2006a).

### Molecular dynamic simulation (MD)

The ligand–protein complex was selected, and the system was built by using a system builder module of orthorhombic box shape with the size of 10*10*10 Å and then predefined SPC (simple point charge) solvent model, OPLS3 as a force field was selected, For all system required number of  sodium/chloride ions were added to neutralize by maintaining the salt concentration of 0.15 M (Na + and Cl-). The built system was minimized to relax a model system into a local energy minimum for 100 ps simulation time. The minimized system was used for performing the MD simulation for 100 ns using NPT (constant temperature and constant pressure ensemble) ensemble class at 300 K temperature and 1.01325 bar pressure. After performing MD simulation, thermal MM-GBSA was performed using trajectory generated from MD simulation for the protein–ligand complex. The MD simulation results were analyzed by generating the simulation interaction diagram (Bowers et al. 2006).

## Result

### Analyses of SARS-CoV-2 and ACE2 receptor-interacting residues

**Fig. 2:**
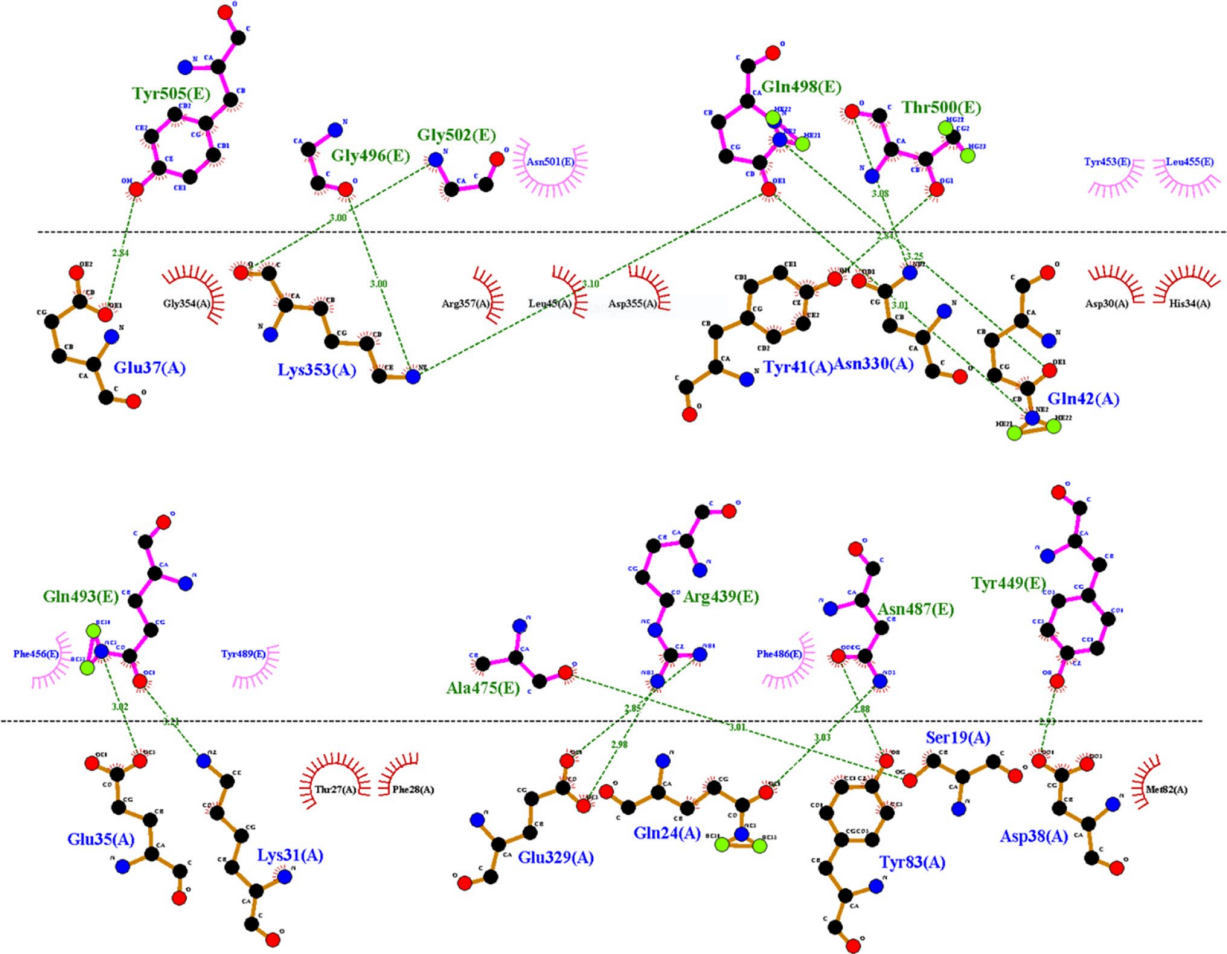
Dimplot of protein complex of SARs-Cov-2 chimeric receptor-binding domain and angiotensin-converting enzyme 2

To enter SARS-CoV-2 into the cell, the receptor-binding domain of SARS-CoV-2 should interact with the ACE2 receptor. The interaction between the ACE2 receptor and SARS-CoV-2 RBD was analyzed by dimplot using LigPlot software (Laskowski and Swindells 2011). From the plot, all amino acid pairs which form a hydrogen bond (H-bond) between chain A and E are represented in Fig. 2. In his study, these interacting residues were targeted to inhibit the entry of the SARS-CoV-2 using the database created from selected Ayurvedic and Siddha formulations.

### Docking site identification using the sitemap

To determine the best site for the docking of the ligands, a sitemap analysis of the protein complex was done. The five best-docking sites were determined, the Dscore and site score are shown in Table 1. Among these sites, the one with a Dscore (1.051) and site score more than 1 (site 1–1.018) covering the amino acids involved in hydrogen bond formation between the protein complex as analyzed by dimplot was selected for grid generation and docking of the selected molecules.

**Table 1:** Sitemap analyses for protein complex containing SARS-CoV-2 chimeric receptor-binding domain and angiotensin-converting enzyme 2

Title	Site score	Dscore	Volume	Residues
Chain A & E site 3	1.047	1.069	470.93	Chain A: 276,279,288,289,290,291,292,294,346,365,366,367,370,371,374,375,406,409,410,413,428,434,437,438,441,442,445,446,449,515,518,519,522
Chain A & E site 2	1.027	1.048	636.26	Chain A: 85,90,91,92,94,95,98,99,101,102,103,104,107,193,194,195,196,202,205,206,208,209,210,211,212,219,391,392,396,397,560,561,562,563,564,565,566,569,714
Chain A & E site 1	1.018	1.051	968.63	Chain A: 26,29,30,32,33,34,35,37,38,40,70,73,74,77,92,93,95,96,99,100,102,103,104,105,106,324,346,347,349,350,352,353,354,355,356,375,378,382,385,386,387,389,390,391,392,393,394,398,401,402,505,510,514,515 Chain E: 403,405,406,408,409,416,417,453,493,494,495,496,497,501,502,503,504,505
Chain A & E site 5	0.781	0.804	137.88	Chain E: 335,336,337,338,339,340,342,343,344,347,364,365,367,368,371,373,374,436,437,438,440,441,509
Chain A & E site 4	0.744	0.626	102.21	Chain E: 454,456,457,458,459,467,469,471,472,473,474,480,482,491

### Ligand docking

Database of ligands created using selected Ayurvedic and Siddha formulation were docked using the extra precision docking protocol to obtain a correlation between a good pose of drugs and a high dock score. The top 10 ligands showing interaction with the amino acids analyzed by dimplot were selected and are listed in Table 2. The docking score of the standard drugs favipiravir and ribavirin had docking scores of − 7.301 and − 4.807 kcal/mol, respectively. The docking score of the top ten ligands ranged from − 8.79 to − 4.86 kcal/mol. Among them, iso-chlorogenic acid ( − 8.79 kcal/mol) showed the highest docking score by interacting with protein complex by forming a hydrogen bond with Gly E 496, Val E 417, Asp E 406, His A 34, and salt bridge with Arg E 408.

**Table 2:** 2D interaction diagrams of top ten selected ligands with a summary of Docking score, glide energy and MM-GBSA dG bind and all non-bonding interactions

Sl No.	Name	PubChem CID	Docking score	Glide energy	MM-GBSA dg bind	Non-bonding interaction	Interaction diagram
1.	Favipiravir	492,405	− 7.301	− 45.357	− 36.3	H-bond: Phe E 497, Tyr E 453, Tyr E 505. Hydrophobic interaction Charged negative: Asp A 38. Charged positive: Lys A 353, Lys E 403. Hydrophobic: Tyr E 495. Polar: Gln E 498, Gln E 493, Ser E 494, Asn E 501, His A 34	
2.	Ribavirin	37,542	− 4.807	− 29.542	− 6.97	H-bond: Gly E 496, His A 34, Tyr E 453, Tyr E 505. Hydrophobic interaction Charged negative: Glu A 37, Asp A 38 Charged positive: Lys A 353, Lys E 403, Arg A 393. Hydrophobic: Tyr E 495, Phe E 497. Polar: Asn E 501, Gln E 506, Gln E 498, Gln E 493, Ser E 494, Asn A 33	
3.	Iso-chlorogenic	6,436,237	− 8.799	− 44.248	− 18.01	H-bond: Gly E 496, Val E 417, Asp E 406, His A 34 Salt bridge: Arg E 408. Hydrophobic interaction. Charged negative: Asp E 405, Glu A 37, Asp A 38. Charged positive: Lys A 353, Lys E 403. Hydrophobic: Ile E 418, Tyr E 495, Tyr E 505, Tyr E 449, Tyr E 453. Polar: Gln E 409, Gln E 493, Ser E 494	
4.	Taxiphyllin	107,721	− 7.595	− 41.558	− 36.29	H-bond: Arg A 393, Ala A 348, Ash A 350. Hydrophobic interaction Charged negative: Glu A 37, Asp A 382 Charged positive: Lys A 353 Hydrophobic: Trp A 349, Leu A 351, Phe A 40, Tyr A 385, Phe A 390 Polar: Hie 401, Asn A 394	
5.	Vasicine	72,610	− 6.985	− 26.254	− 40.77	H-bond: Arg A 393, Asn A 394 Pi cation: Phe A 390, Phe A 40. Hydrophobic interaction Charged negative: Ash A 350, Glu A 37 Charged positive: Lys A 353 Hydrophobic: Leu A 391, Tyr A 385, Leu A 351	
6.	( +)-Catechin	9064	− 6.915	− 40.093	− 35.15	H-bond: Leu A 391, Arg A 393, Ash A 350.Hydrophobic interaction Charged positive: Lys A 353 Hydrophobic: Phe A 40, Leu A 351, Phe A 390, Ala A 99, Leu A 73, Trp A 69 Polar: Asn A 394	
7.	Caffeic Acid	689,043	− 5.563	− 26.482	− 4.63	H-bond: Gly E 496. Salt bridge: Lys E 403 Hydrophobic interaction Charged negative: Asp E 406, Glu A 37, Asp A 38 Charged positive: Lys A 353 Hydrophobic: Tyr E 453, Tyr E 505, Tyr E 495, Phe E 497 Polar: His A 34	
8.	2-Methyl-3-Methylene-1,4-Dioxane	287,960	− 5.403	− 17.555	− 23.56	H-bond: Gly E 496 Hydrophobic interaction Charged negative: Glu A 37, Asp A 38 Charged positive: Lys E 403, Lys A 353. Hydrophobic: Tyr E 453, Tyr E 505, Tyr E 449, Tyr E 495 Polar: His A 34, Gln E 493, Ser E 494	
9.	Furaneol	19,309	− 5.364	− 24.079	− 28.6	H-bond: Lys E 403, Gly E 496 Hydrophobic interaction Charged negative: Glu A 37, Asp A 38 Charged positive: Lys A 353 Hydrophobic: Tyr E 453, Tyr E 505, Tyr 449, Tyr 495, Phe E 497 Polar: His A 34, Gln E 493, Ser E 494, Asn E 501	
10.	Chrysoeriol	5,280,666	− 5.325	− 30.439	− 34.36	H-bond: Asn A 394, Arg A 393, Trp A 69 Pi–Pi stacking: Phe A 40, Phe A 390 Hydrophobic interaction Charged negative: Glu A 37, Ash A 350 Charged positive: Lys A 353 Hydrophobic: Leu A 73, Tyr A 385, Leu A 351, Leu A 391	
11.	2-Methoxy-4-	332	− 5.289	− 26.672	− 32.7	H-bond: Gly E 496 Hydrophobic interaction Charged negative: Asp E 406, Glu A 37, Asp A 38 Charged positive: Lys E 403, Lys A 353 Hydrophobic: Phe E 497, Tyr E 495, Tyr E 449, Tyr E 505 Polar: His A 34, Asn E 501, Gln E 493	
12.	Eugenol	3314	− 4.867	− 26.837	− 31.14	Hydrophobic interaction Charged negative: Glu A 37, Asp A 38, Asp E 406 Charged positive: Lys E 403, Lys A 353 Hydrophobic: Tyr E 453, Tyr E 495, Phe E 497, Tyr E 505, Tyr E 449 Polar: His A 34, Gln E 493, Ser E 494, Gln E 498, Asn E 501	

### Free ligand binding energy calculation

After docking, the top 10 molecules selected based on docking score and interaction with residues, which are essential for binding ACE2 to SARS-CoV-2, were further analyzed for binding energy by MM-GBSA. The binding free energy of the standard drugs favipiravir and ribavirin was − 36.3 and − 6.97 kcal/mol. Ribavirin had the lowest binding energy among all the selected ligands. The free ligand binding energy of all selected ligands ranged from − 18.01 to − 40.77 kcal/mol, highest shown by Vasicine. All ligands except caffeic acid and iso-chlorogenic acid had binding energy of more than -20 kcal/mol.

### ADME analysis

ADME properties of the selected ligands were predicted by using the Qikprop module. The assessment was done using various descriptor calculations as tabulated in Table 3 like QPlogPo/w, QPlogS, QPPCaco, QPlogHERG, % human oral absorption, PSA, and Lipinski rule of five. All ligands did not violate the rule of five, showing drug-like property. All selected ligands have good aqueous solubility, hydrophobic and hydrophilic balance as predicted by QPlogS and QPLogPo/w vales, respectively. The QPPCaco and % oral absorption of all molecules except iso-chlorogenic acid were within the acceptable range. The QlogHERG values were more than -5; therefore, ligands have not shown the potential to inhibit the hERG potassium channel.

**Table 3:** ADME prediction of the top ten selected ligands by using various parameters like solubility, partition, toxicity, absorption and draggability

Sl No.	Name	QPlogPo/w	QPlogS	QPlogHERG	QPPCaco	% Human Oral Absorption	PSA	Rule of Five
1.	Iso-chlorogenic acid	− 0.301	− 2.218	− 2.914	1.979	17.531	184.001	1
2.	Taxiphyllin	− 1.499	− 0.539	− 4.75	30.763	44.801	150.57	0
3.	Vasicine	1.892	− 2.465	− 4.083	2398.587	100	37.543	0
4.	( +)-Catechin	0.448	− 2.662	− 4.847	49.179	59.848	117.119	0
5.	Caffeic Acid	0.545	− 1.293	− 2.169	22.354	54.287	95.571	0
6.	2-Methyl-3-Methylene-1,4-Dioxane	0.767	− 0.233	− 2.386	9906.038	100	16.996	0
7.	Furaneol	0.225	− 0.825	− 2.715	1104.367	82.727	60.06	0
8.	Chrysoeriol	1.761	− 3.633	− 5.058	115.81	74.193	107.153	0
9.	2-Methoxy-4-Vinylphenol	1.908	− 1.623	− 3.749	3043.607	100	29.937	0
10.	Eugenol	2.661	− 2.387	− 3.954	3043.414	100	29.952	0

### Induced fit docking (IFD)—SP

**Fig. 3:**
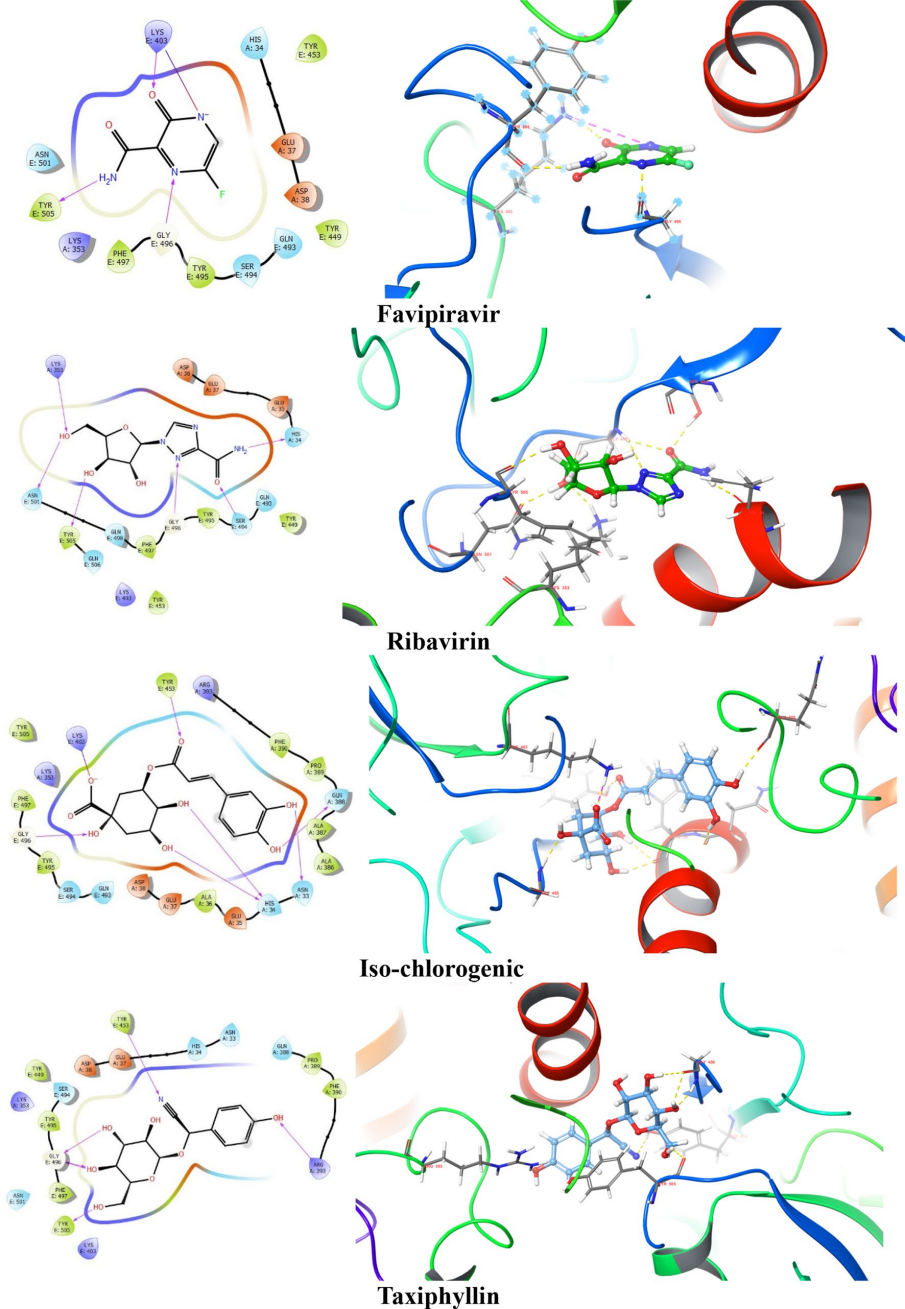

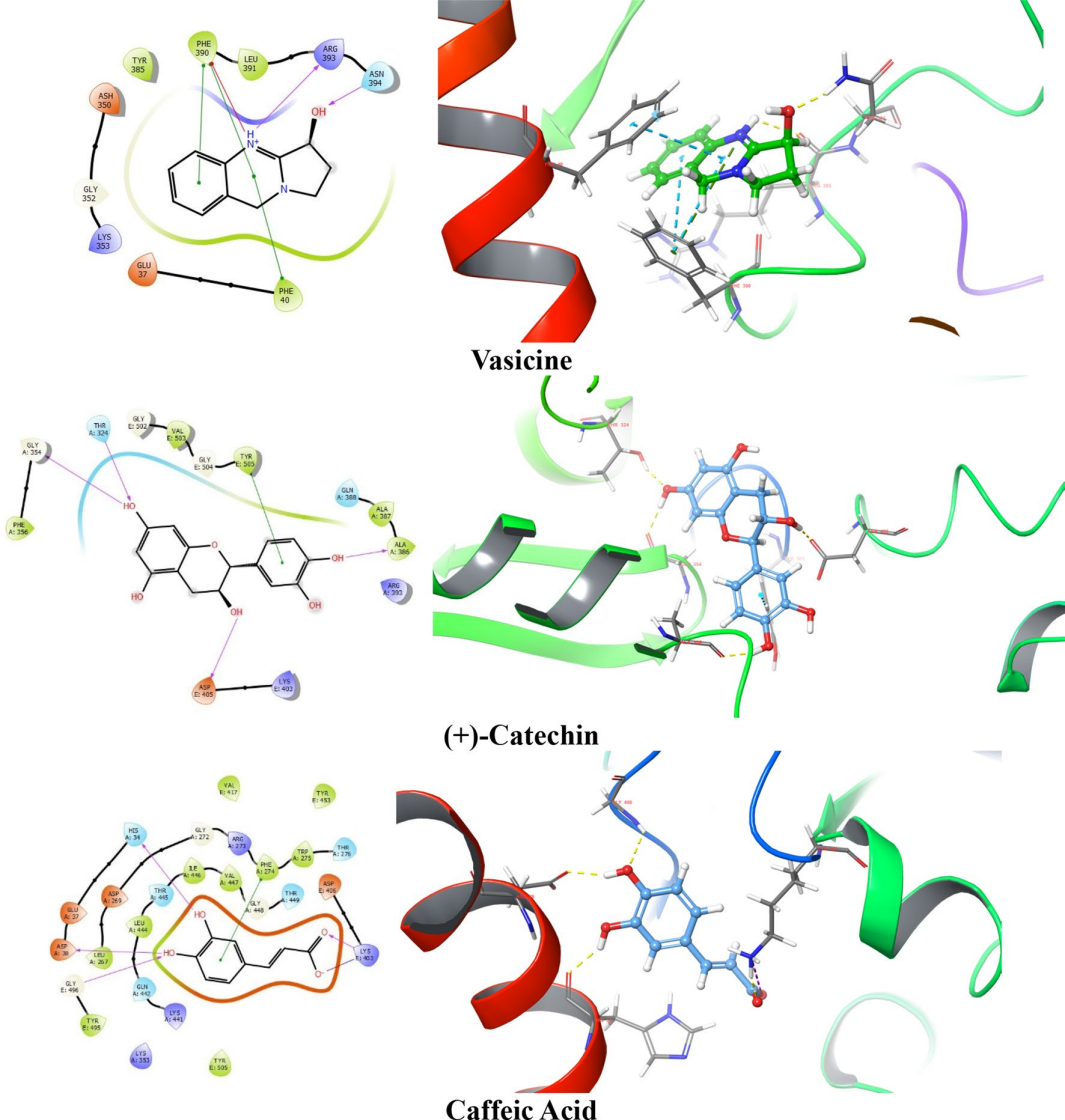
2D and 3D interaction diagram of favipiravir, ribavirin, iso-chlorogenic, taxiphyllin, vasicine, ( +)-catechin and caffeic acid with the protein complex of SARs- CoV-2 RBD and ACE 2 showing the hydrogen bond (represented by yellow dotted line) and Pi–Pi stacking

After analyzing the docking score, glide energy, dG bind score, ADME, and the interactions with the protein, top 5 ligands, and standard drugs were further studied by using Induced fit docking protocol. IFD analysis involves flexible docking that helps to confirm whether there are any changes in the binding of ligands at different poses with the amino acid residues of the target protein complex. Maximum 20 possible poses were generated for favipiravir, ribavirin, iso-chlorogenic acid, taxiphyllin, vasicine, catechin, and caffeic acid by providing flexibility to the ligand and amino acid residue of the protein complex. Further, ligand interaction with the key residues was analyzed for selecting pose for molecular dynamics. The 2D and 3D ligand interactions obtained after IFD are represented in Fig. 3. There were many differences in interaction seen in the XP docking pose and IFD pose, which is highlighted in Table 4.

**Table 4:** Summary of difference between interaction of ligands with protein complex in XP docking and induced fit docking

Ligands	Interaction shown in XP docking	Interaction shown in induced fit docking
Favipiravir	H-bond: **Phe E 497, Tyr E 453**, Tyr E 505. Hydrophobic interaction Charged negative: Asp A 38. Charged positive: Lys A 353, **Lys E 403.** Hydrophobic: Tyr E 495. Polar: **Gln E 498**, Gln E 493, Ser E 494, Asn E 501, His A 34	H-bond: **Gly E 496, Lys E 403**, Tyr E 505. Salt bridge: **Lys E 403.** Hydrophobic interaction Charged negative: Asp A 38, **Glu A 37**. Charged positive: Lys A 353. Hydrophobic: Tyr E 495, **Tyr E 449, Tyr E 453, Phe E 497.** Polar: **Asn E 501**, Gln E 493, Ser E 494, Asn E 501, His A 34
Ribavirin	H-bond: **Gly E 496,** His A 34, **Tyr E 453**, Tyr E 505. Hydrophobic interaction Charged negative: Glu A 37, Asp A 38. Charged positive: Lys A 353, Lys E 403, **Arg A 393.** Hydrophobic: Tyr E 495, Phe E 497. Polar: **Asn E 501**, Gln E 506, Gln E 498, Gln E 493, Ser E 494, **Asn A 33**	H-bond: **Ser E 494**, **Asn E 501**, Tyr E 505, **Lys A 353**, His A 34. Hydrophobic interaction Charged negative: Glu A 37, Asp A 38, **Glu A 35**. Charged positive: Lys A 353, Lys E 403. Hydrophobic: Tyr E 495, Phe E 497, **Tyr E 449, Tyr E 453**. Polar: Gln E 506, Gln E 498, Gln E 493
Iso-chlorogenic	H-bond: Gly E 496, **Val E 417**, **Asp E 406**, His A 34 Salt bridge: **Arg E 408.** Hydrophobic interaction Charged negative: **Asp E 405**, Glu A 37, Asp A 38. Charged positive: Lys A 353, **Lys E 403.** Hydrophobic: **Ile E 418**, Tyr E 495, Tyr E 505, **Tyr E 449, Tyr E 453.** Polar: **Gln E 409**, Gln E 493, Ser E 494	H-bond: **Asn A 33**, **Gln A 388**, **Tyr E 453**, His A 34, **Lys E 403,** Gly E 496. Salt bridge: **Lys E 403**. Hydrophobic interaction Charged negative: **Glu E 35**, Glu A 37, Asp A 38. Charged positive: Lys A 353, **Arg A 393**. Hydrophobic: **Phe A 390**, **Pro A 389**, **Ala A 387**, **Ala A 386**, **Ala A 36**, Tyr E 495, **Phe E 497**, Tyr E 505. Polar: Gln E 493, Ser E 494
Taxiphyllin	H-bond: Arg A 393, **Ala A 348**, **Ash A 350.** Hydrophobic interaction Charged negative: Glu A 37, **Asp A 382.** Charged positive: Lys A 353. Hydrophobic: **Trp A 349, Leu A 351, Phe A 40, Tyr A 385,** Phe A 390. Polar: **Hie 401,** Asn A 394	H-bond: Arg A 393, **Tyr E 505**, **Tyr E 453, Gly E 496.** Hydrophobic interaction Charged negative: Glu A 37, **Asp A 38.** Charged positive: Lys A 353, **Lys E 403.** Hydrophobic: Phe A 390, **Tyr E 449, Tyr E 495**, **Phe E 497**, **Pro A 389**. Polar: **Gln A 388, Asn A 33, His A 34**, Ser E 494, **Asn E 501**
Vasicine	H-bond: Arg A 393, Asn A 394 Pi cation: Phe A 390, Phe A 40. Hydrophobic interaction Charged negative: Ash A 350, Glu A 37 Charged positive: Lys A 353 Hydrophobic: Leu A 391, Tyr A 385, **Leu A 351**	H-bond: Arg A 393, Asn A 394Pi cation: Phe A 390, Phe A 40.Pi–Pi stacking: **Phe A 390**Hydrophobic interaction Charged negative: Ash A 350, Glu A 37 Charged positive: Lys A 353 Hydrophobic: Leu A 391, Tyr A 385
( +)-Catechin	H-bond: **Leu A 391**, **Arg A 393**, **Ash A 350**. Hydrophobic interaction Charged positive: **Lys A 353.** Hydrophobic: **Phe A 40, Leu A 351, Phe A 390, Ala A 99, Leu A 73, Trp A 69.** Polar: **Asn A 394**	H-bond: **Ala A 386, Thr A 324, Gly A 354, Asp E 405.** Pi–Pi stacking**: Tyr E 505.** Hydrophobic interaction Charged positive: **Lys E 403, Arg A 393.** Hydrophobic: **Ala A 387, Val E 503, Phe A 356.** Polar: **Gln A 388**
Caffeic Acid	H-bond: Gly E 496.Salt bridge: Lys E 403 Hydrophobic interaction Charged negative: Asp E 406, Glu A 37, **Asp A 38.** Charged positive: Lys A 353 Hydrophobic: Tyr E 453, Tyr E 505, Tyr E 495, **Phe E 497** Polar: His A 34	H-bond: **His A 34, Asp A 38**, Gly E 496, **Lys E 403** Salt bridge: Lys E 403 Pi–Pi stacking: **Phe A 274.**Hydrophobic interaction Charged negative: Asp E 406, Glu A 37, **Asp A 269** Charged positive: Lys A 353, **Lys A 441, Arg A 273.** Hydrophobic: Tyr E 453, Tyr E 505, Tyr E 495, **Leu A 267, Leu A 444, Ile A 446, Val A 447, Trp A 275, Val E 471** Polar: **Gln A 442, Thr A 445, Thr A 276, Thr A 449**

Favipiravir showed H-bond with Gly E 496 in the IFD pose, which is one of the key residues, in addition to retaining Tyr E 505. Ribavirin retained H-bond interaction with Tyr E 505 and formed H-bond with Lys A 353. For Iso-chlorogenic acid, no new interaction with key residue was formed, and H-bond interaction with Gly E 496 was retained. Taxiphyllin formed H-bond interaction with Tyr E 505, Gly E 496, which are essential for binding to ACE2. In the case of Vasicine, no interaction with any key residue was seen even in the IFD pose. Catechin had not shown any important interaction in the XP docked pose, but in the IFD pose, it showed Pi–Pi stacking type of interaction with Tyr E 505. Caffeic acid showed new H-bond interaction with Asp A 38 retaining Gly E 496. This change in the interaction between XP docking pose and IFD pose of the ligands may be due to the mobility of the protein amino acid chains, which is not seen in the XP docking; hence the pose showing interaction with key residues were selected for MD docking studies.

### Molecular Dynamics (MD) simulation analysis of the compounds with thermal

#### MM-GBSA

**Fig. 4:**
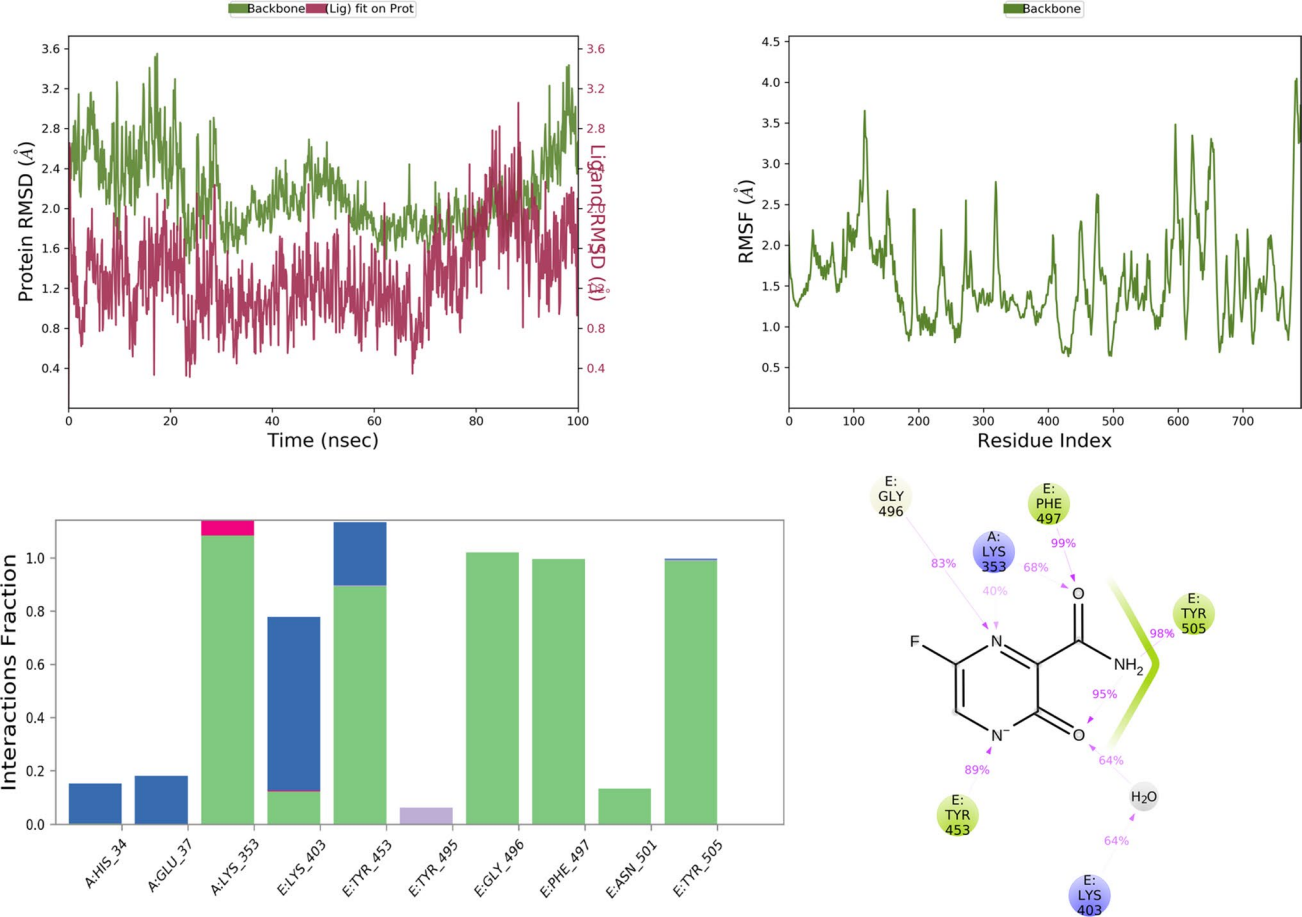
RMSD, RMSF, and protein–ligand contact plots of favipiravir with protein complex of SARS-CoV-2 chimeric receptor-binding domain and angiotensin-converting enzyme 2 observed during MD simulation

**Fig. 5:**
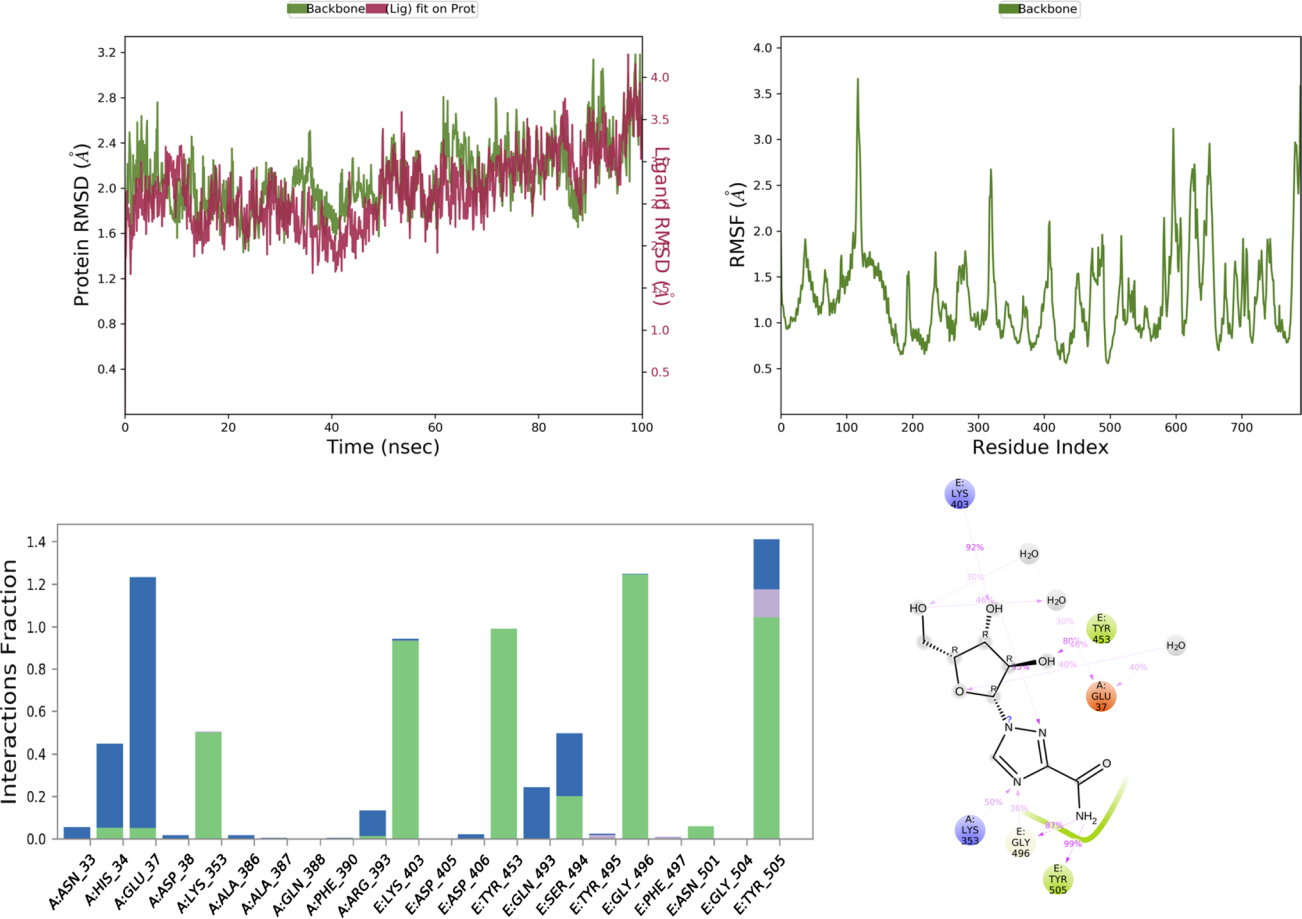
RMSD, RMSF, and protein–ligand contact plots of ribavirin with protein complex of SARS-CoV-2 chimeric receptor-binding domain and angiotensin-converting enzyme 2 observed during MD simulation

**Fig. 6:**
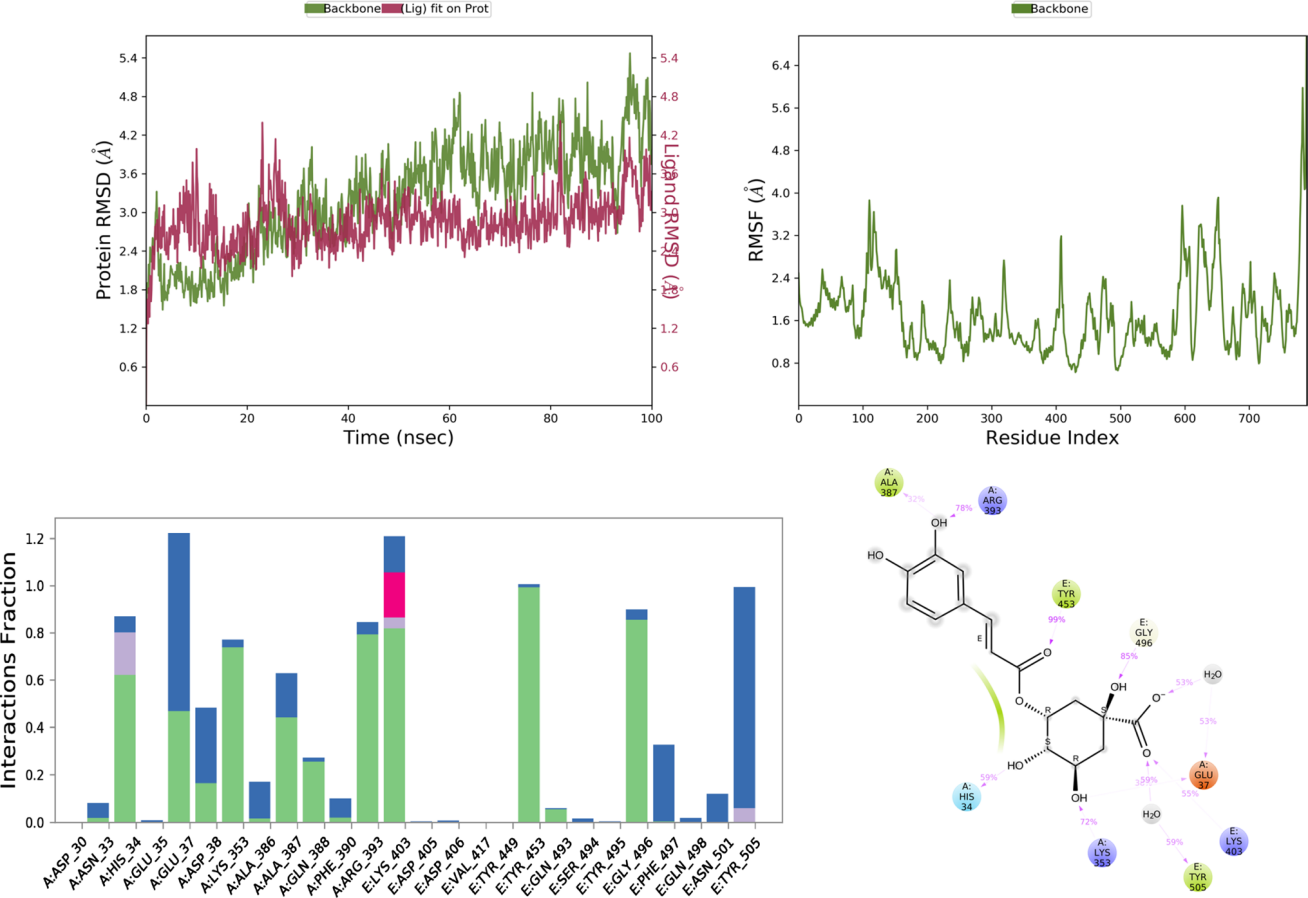
RMSD, RMSF, and protein–ligand contact plots of iso-chlorogenic acid with protein complex of SARS-CoV-2 chimeric receptor-binding domain and angiotensin-converting enzyme 2 observed during MD simulation

**Fig. 7:**
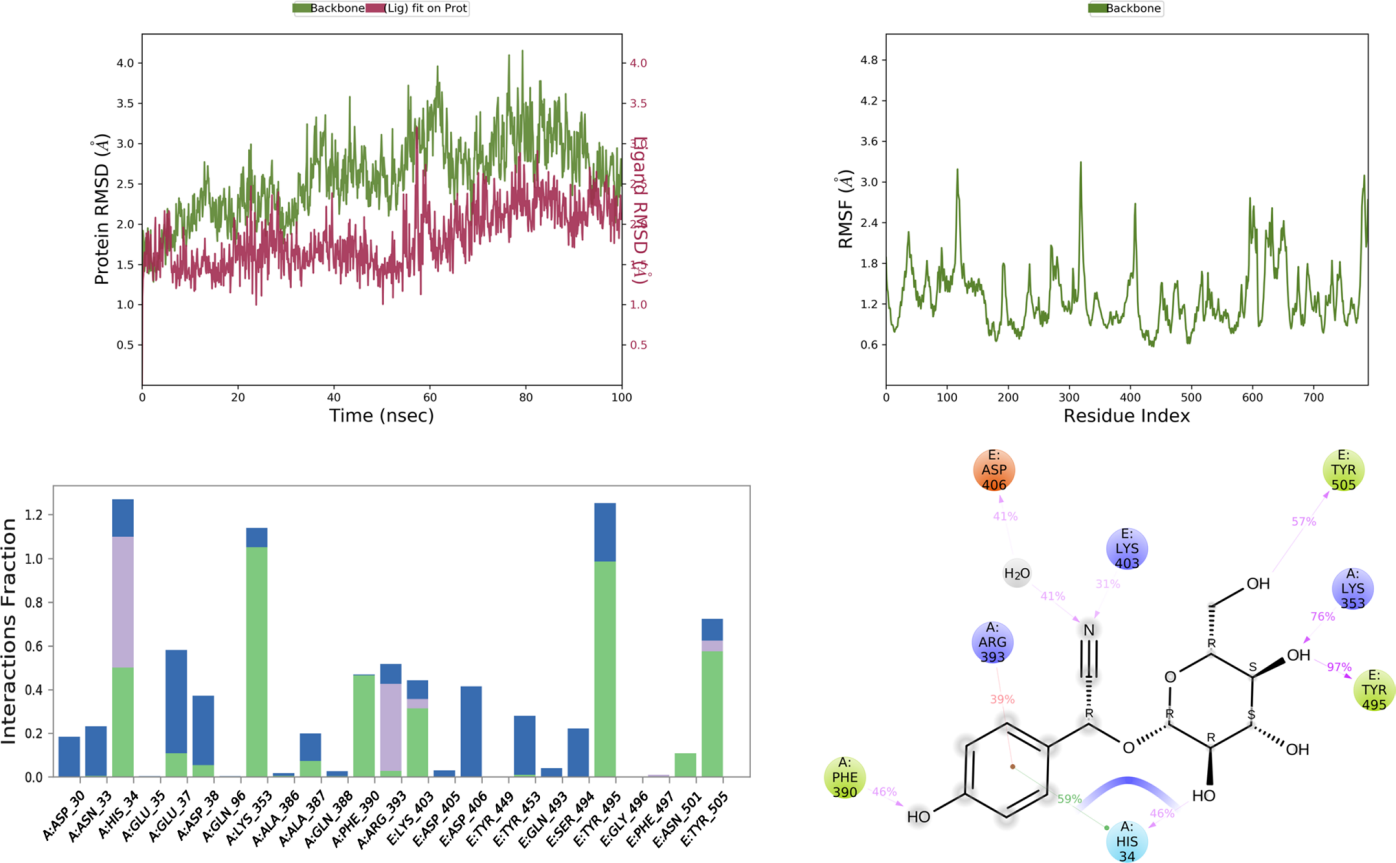
RMSD, RMSF, and protein–ligand contact plots of taxiphyllin with protein complex of SARS-CoV-2 chimeric receptor-binding domain and angiotensin-converting enzyme 2 observed during MD simulation

**Fig. 8:**
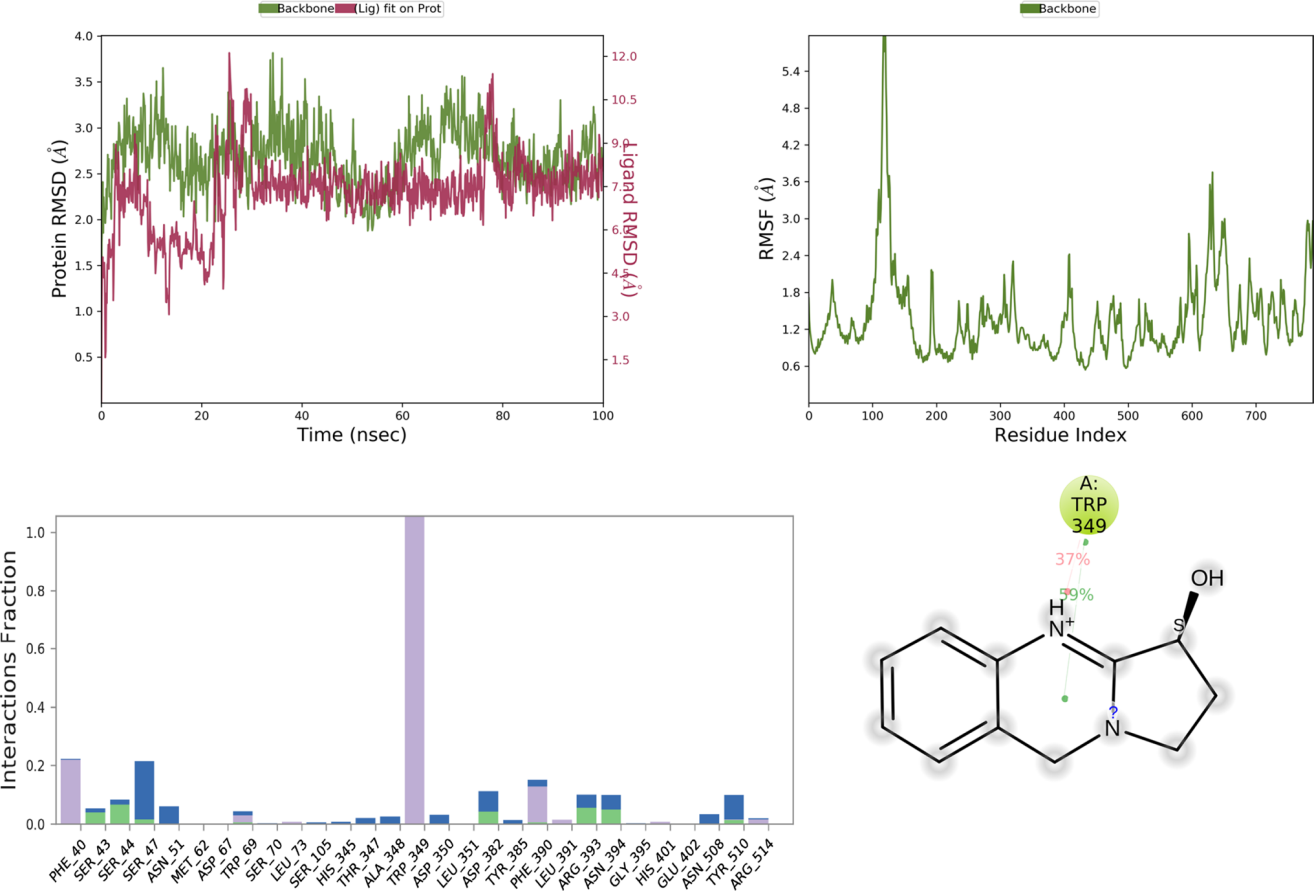
RMSD, RMSF, and protein–ligand contact plots of vasicine with protein complex of SARS-CoV-2 chimeric receptor-binding domain and angiotensin-converting enzyme 2 observed during MD simulation

**Fig. 9:**
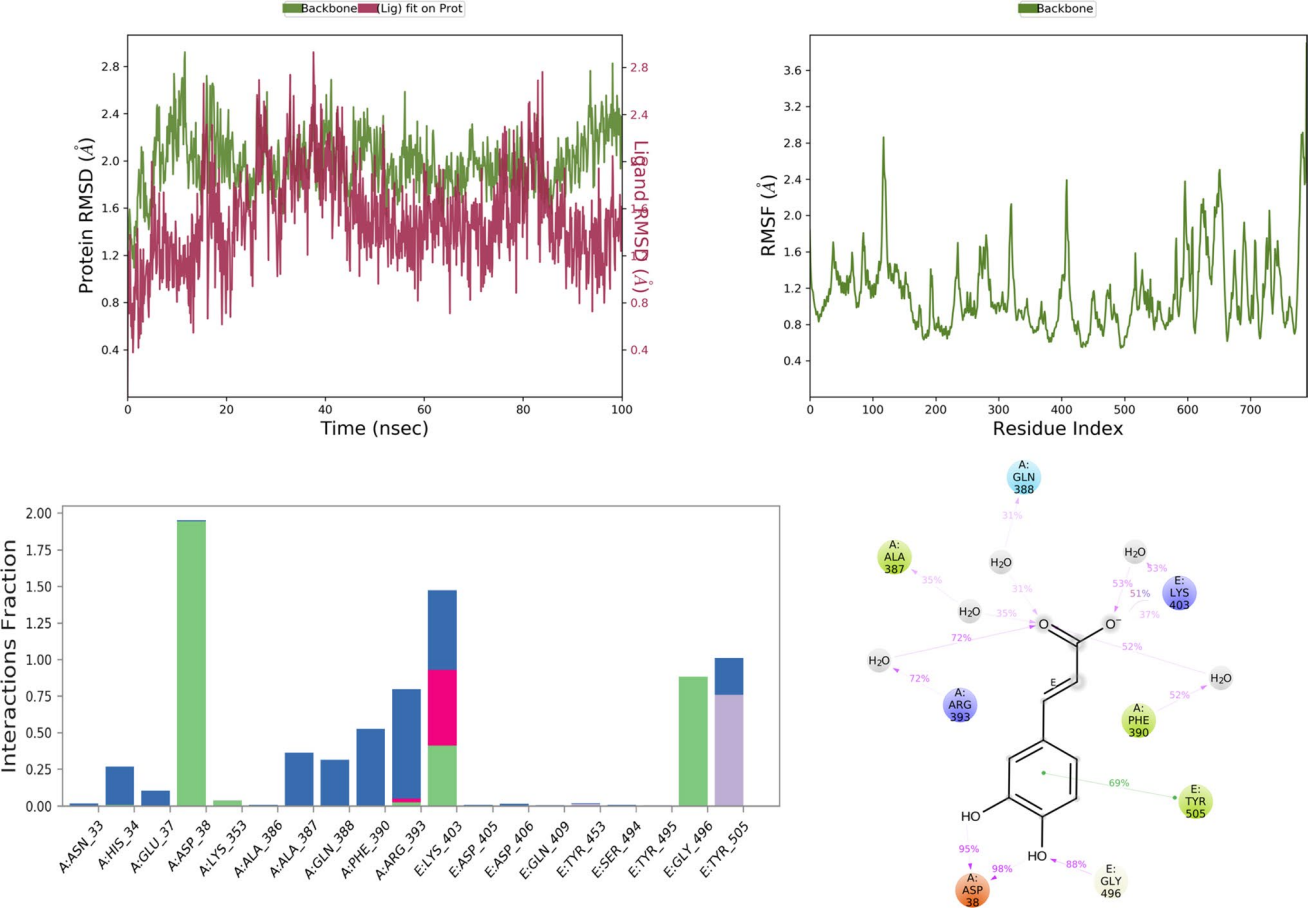
RMSD, RMSF, and protein–ligand contact plots of ( +)-catechin with protein complex of SARS-CoV-2 chimeric receptor-binding domain and angiotensin-converting enzyme 2 observed during MD simulation

**Fig. 10:**
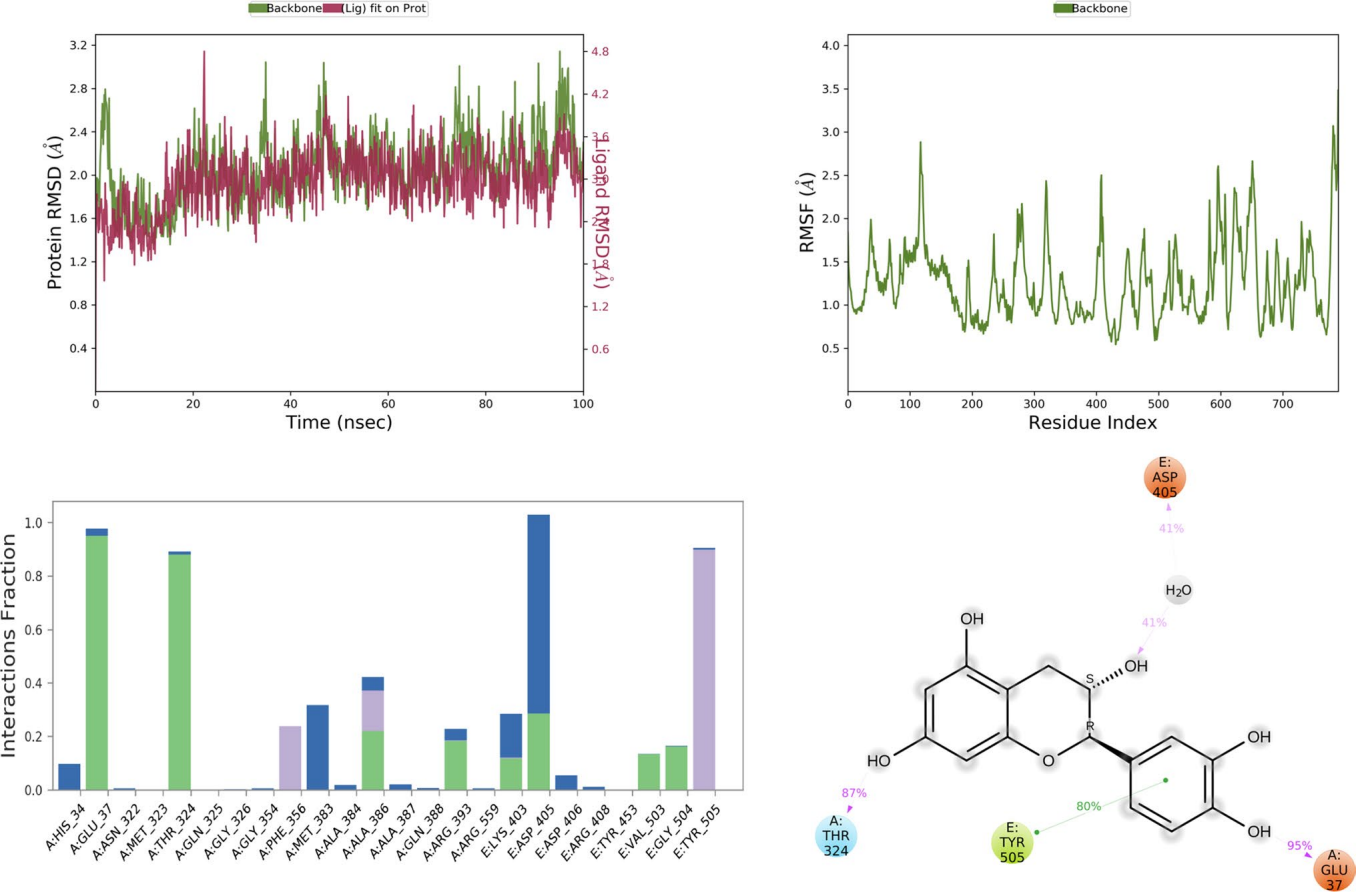
RMSD, RMSF, and protein–ligand contact plots of caffeic acid with protein complex of SARS-CoV-2 chimeric receptor-binding domain and angiotensin-converting enzyme 2 observed during MD simulation

MD simulation study mimics the physiological condition, thereby, providing information related to protein–ligand interactions with biological significance. Currently, MD simulation was done for standard drugs and selected top five docking score favipiravir, ribavirin, iso-chlorogenic acid, taxiphyllin, vasicine, catechin, and caffeic acid. Results of the MD simulation are represented in Figs. 4, 5, 6, 7, 8, 9, and 10 in order of name of ligands mentioned above.

The RMSD plot analysis of the MD simulation showed that protein structure in all the simulations was stable with RMSD less than 5 Å. Still, after analyzing ligand RMSD, it was found that all ligands except vasicine (ligand RMSD 0.5–12 Å) were stable during molecular simulations, which means vasicine was not able to form a stable interaction with the protein (Table 5).

**Table 5:** Thermal MM-GBSA analysis of the top five ligands iso-chlorogenic acid, taxiphyllin, vasicine, catechin, and caffeic acid and standard drug molecules

Sl No.	Name	Average binding energy in Kcal/mol	Maximum binding energy among all frame in Kcal/mol
	Favipiravir	− 33.15	− 41.61
	Ribavirin	− 49.80	− 61.84
	Iso-chlorogenic acid	− 46.68	− 82.24
	Vasicine	1973.84	− 57.61
	Taxiphyllin	− 44.57	− 63.28
	Catechin	− 47.28	− 55.14
	Caffeic acid	− 28.54	− 37.63

Favipiravir bound to the protein exhibited a combination of hydrogen bond, water bridge, and bonding interactions. Among them, it showed H-bond interaction with key residues like Tyr E 505 and Gly E 496 for more than 80% of the time of MD simulation, as seen in Fig. 4. Ribavirin showed interaction with Tyr E 505 (99%), Glu A 37 (40%), Lys A 353 (50%), Gly E 496 (87%), for Tyr E 505, Gly E 496, Lys A 353 it was mostly H-bond mediated interaction, but for Glu A 37 it was water bridge mediated interaction as seen in Fig. 5.

Among all test ligands, iso-chlorogenic acid showed the maximum number of interactions with the protein. Interactions with key residue similar to ribavirin like Tyr E 505, Glu A 37, Lys A 353, Gly E 496 were present. Still, the type of interactions was different, Tyr E 505 was mostly water bridge (59%) mediated interactions, Glu A 37 was both water bridge (53%) and H-bond mediated (38%) interactions, Gly E 496, Lys A 353 were H-bond mediated for 85% and 72% of the time, respectively, as seen in Fig. 6. Though the taxiphyllin–protein complex was very stable, it formed interaction with only two key residues Tyr E 505, Lys A 353; both were mostly H-bond types of interaction for 57% and 76% of the time as seen in Fig. 7. Vasicine didn't show interaction with any of the key residues, and the protein–ligand complex was also not stable, as shown in Fig. 8. Catechin showed interactions with Tyr E 505 for 69% of the time, which was the hydrophobic type; Glu A 38 showed H-bond type interaction with two different hydroxyl groups of catechin and Gly E 496 showed H-bond interaction for 88% of the time as seen in Fig. 9. Caffeic acid interacted with only two amino acids Tyr E 505 (hydrophobic) and Gly A 37 (H-bond), for a period of 80% and 95% of the time, as shown in Fig. 10.

Thermal MM-GSBA was performed using the trajectory generated after MD simulation. The average binding energy of all the ligands was more than -25 kcal/mol except vasicine (1973.84 kcal/mol), which showed positive value proving that the protein–ligand complex was not stable. The most stable complex among all was ribavirin with − 49.8 kcal/mol. Iso-chlorogenic acid, which showed the maximum number of interactions with key residue, had average binding energy of − 46.68 kcal/mol.

## Discussion

In this work, an *in silico* approach is used to evaluate the traditional formulation of Siddha and Ayurveda system of medicine like Kabasura Kudineer, Shwas Kuthar Rasa with Kantakari and Pippali churna, Talisadi churna selected from the advisory issued by the ministry of Ayush for management of COVID-19 pandemic. After performing docking, ADME prediction, free binding energy determination, we narrowed it down to the top five phytoconstituents iso-chlorogenic acid, taxiphyllin, vasicine, catechin, and caffeic acid. On a literature survey, it was found that vasicine has shown activity against Influenza Virus Infection (Chavan et al. 2013). Iso-chlorogenic acid has a potent anti-hepatitis B activity (Hao et al. 2012), and antienterovirus (Cao et al. 2017), one of its derivate iso-chlorogenic acid A has been proven by the *in vitro* study to inhibit viral entry into the cell at 100 micromolar concentration (Zhang et al. 2021). Catechin inhibits both Mpro and spike protein of SARS-CoV-2 predicted by using computational study (Srivastava et al. 2020). Caffeic acid was able to impair the binding interaction of human coronavirus NL63 (HCoV-NL63) with ACE2 receptor (IC50 = 3.54 μM) (Weng et al. 2019). In this study, we have tried to identify phytoconstituents showing better binding efficacy with S-protein RBD complex with ACE2 and to compare its binding with already approved drugs like ribavirin and favipiravir. MD simulation and thermal MM-GBSA were performed. By MD, we got to know that iso-chlorogenic acid showed stable interaction with the key residues like that of the ribavirin and had RMSD in the acceptable range, which was further confirmed by the thermal MM-GBSA result proving that iso-chlorogenic acid forms a more stable complex with the target protein. All other top five phytoconstituents except vasicine formed stable interaction with key residues as discussed in results showing that many phytoconstituents might be able to inhibit the entry of the virus into the cell. When compared to other similar studies published recently with PDB ID 6VW1, we found that (Morgon et al. 2021) showed that (S)-Linezolid had a binding affinity of − 8.05 kcal/mol with no interaction with key residues. However, in our study iso-chlorogenic acid ( − 8.799 kcal/mol) had interaction with key residues with higher binding affinity. In another study by Junaid et al. (2021), docking of Vitamin B, C, D was done and the binding energy − 9.18, − 12.75, − 7.92, respectively, was reported, interaction with key residue His A 353 was only observed for vitamin C.

This provides molecular bases for using the traditional Siddha- and Ayurvedic-based classical formulations for treating COVID-19. It also helps the researcher develop more potent molecules that can be used to control the spreading of the pandemic and mass-produce the drug molecule. Therefore, we made a small effort to identify the molecule which can inhibit the entry of COVID-19 through ACE2 receptor into the cell by using phytoconstituents present in Ayurvedic and Siddha formulation advised by the Ministry of Ayush.

## Conclusion

The spike protein of COVID-19 has been proven to assist the entry of the virus into the cell more easily compared to its counterpart in the family of the SARS virus. Inhibition of binding of spike protein to the ACE2 of the host cell is one of the best possible approaches which can be used to prevent the spreading of the infection by COVID-19. In this study, we have used *in silico* molecular docking studies for the 133 phytoconstituents selected from Ayurvedic and Siddha formulation against the protein of SARS-CoV-2 RBD complexed with ACE2 receptor (PDB ID: 6VW1). The results showed that iso-chlorogenic has a similar binding affinity and high binding interactions with the amino acid residues involved in binding of SARS-CoV-2 RBD with the ACE2 receptor compared to favipiravir and ribavirin. Further, *in silico* pharmacokinetic and toxicity prediction has shown poor oral bioavailability and is free from toxicity. Based on this, we can say that iso-chlorogenic acid among all phytoconstituent in the selected formulation has the ability to inhibit the entry of the COVID-19 into the host cell. However, further *In vitro* and *In vivo* studies are required to confirm the findings.

## Supplementary Information

Below is the link to the electronic supplementary material.

Supplementary file1 (DOCX 29 KB)
