# Supplementary material for: Structure-based docking, pharmacokinetic evaluation, and molecular dynamics-guided evaluation of traditional formulation against SARS-CoV-2 spike protein receptor bind domain and ACE2 receptor complex
Source: Chem Zvesti. 2021 Oct 18;76(2):1063–83. doi: 10.1007/s11696-021-01917-z (PMC8522134; doi:10.1007/s11696-021-01917-z)
Supplement: Supplementary file 1 — Supplementary file1 (DOCX 29 KB) [file 11696_2021_1917_MOESM1_ESM.docx]

| Pubchem ID | Name | Docking score | MMGBSA dg Bind |
| --- | --- | --- | --- |
| 72610 | [Vasicine](https://pubchem.ncbi.nlm.nih.gov/compound/72610) | -6.985 | -40.77 |
| 5280343 | [Quercetin](https://pubchem.ncbi.nlm.nih.gov/compound/5280343) | -6.982 | -41.19 |
| 5281697 | [Scutellarein](https://pubchem.ncbi.nlm.nih.gov/compound/5281697) | -6.605 | -33.94 |
| 5280445 | [Luteolin](https://pubchem.ncbi.nlm.nih.gov/compound/5280445) | -5.973 | -38.07 |
| 370 | [Gallic acid](https://pubchem.ncbi.nlm.nih.gov/compound/370) | -5.611 | -16.6 |
| 188323 | [Cirsimaritin](https://pubchem.ncbi.nlm.nih.gov/compound/188323) | -5.584 | -41.04 |
| 5280666 | [Chrysoeriol](https://pubchem.ncbi.nlm.nih.gov/compound/5280666) | -5.325 | -34.36 |
| 462695 | 2-(3,4-Dimethoxyphenyl)-3-hydroxy-5,7-dimethoxy-2,3-dihydro-4H-chromen-4-one | -5.178 | -32.51 |
| 3314 | Eugenol | -4.867 | -31.14 |
| 13963770 | 5-hydroxy-7,8-dimethoxyflavanone | -4.806 | -27.9 |
| 442935 | [Vasicinone](https://pubchem.ncbi.nlm.nih.gov/compound/442935) | -4.708 | -25.37 |
| 5318517 | [Andrographolide](https://pubchem.ncbi.nlm.nih.gov/compound/5318517) | -4.553 | -34.38 |
| 638024 | [Piperine](https://pubchem.ncbi.nlm.nih.gov/compound/638024) | -4.42 | -29.66 |
| 10364 | [Carvacrol](https://pubchem.ncbi.nlm.nih.gov/compound/10364) | -4.404 | -27.57 |
| 5320621 | [Piperlonguminine](https://pubchem.ncbi.nlm.nih.gov/compound/5320621) | -4.379 | -38.12 |
| 11666871 | [Andrograpanin](https://pubchem.ncbi.nlm.nih.gov/compound/11666871) | -4.248 | -21.08 |
| 15215479 | [Tinosponone](https://pubchem.ncbi.nlm.nih.gov/compound/15215479) | -4.19 | -38.04 |
| 72326 | [Betulin](https://pubchem.ncbi.nlm.nih.gov/compound/72326) | -4.158 | -13.43 |
| 222284 | [Beta-sitosterol](https://pubchem.ncbi.nlm.nih.gov/compound/222284) | -4.127 | -36.48 |
| 73337 | [Magnoflorine](https://pubchem.ncbi.nlm.nih.gov/compound/73337) | -3.867 | -9.05 |
| 92138 | [Elemol](https://pubchem.ncbi.nlm.nih.gov/compound/92138) | -3.699 | -21.96 |
| 259846 | [Lupeol](https://pubchem.ncbi.nlm.nih.gov/compound/259846) | -3.607 | -21.97 |
| 638072 | [Squalene](https://pubchem.ncbi.nlm.nih.gov/compound/638072) | -3.567 | -45.69 |
| 194464 | [Bharangin](https://pubchem.ncbi.nlm.nih.gov/compound/194464) | -3.456 | -22.98 |
| 5281437 | [Costunolide](https://pubchem.ncbi.nlm.nih.gov/compound/5281437) | -3.422 | -26.06 |
| 519764 | [Sesquiphellandrene](https://pubchem.ncbi.nlm.nih.gov/compound/519764) | -3.358 | -21.69 |
| 442393 | [Beta-selinene](https://pubchem.ncbi.nlm.nih.gov/compound/442393) | -3.259 | -15.93 |
| 638011 | [Citral](https://pubchem.ncbi.nlm.nih.gov/compound/638011) | -2.948 | -32.81 |
| 5281515 | [Beta-caryophyllene](https://pubchem.ncbi.nlm.nih.gov/compound/5281515) | -2.835 | -24.03 |
| 99856 | [Cyperene](https://pubchem.ncbi.nlm.nih.gov/compound/99856) | -2.461 | -20.83 |
| 403919 | 1-Methyl-4-(6-methylhepta-1,5-dien-2-yl)cyclohex-1-ene | -2.449 | -26.45 |
| 64945 | [Ursolic acid](https://pubchem.ncbi.nlm.nih.gov/compound/64945) | -1.656 | -18.17 |
| 5280794 | [Stigmasterol](https://pubchem.ncbi.nlm.nih.gov/compound/5280794) | -0.972 | -24.98 |
| 73611 | [Solamargine](https://pubchem.ncbi.nlm.nih.gov/compound/73611) | -9.201 | -35.21 |
| 6436237 | [Isochlorogenic Acid](https://pubchem.ncbi.nlm.nih.gov/compound/6436237) | -8.799 | -18.01 |
| 119247 | [Solasonine](https://pubchem.ncbi.nlm.nih.gov/compound/119247) | -8.618 | -34.23 |
| 182630 | Solasurine | -7.925 | -34.96 |
| 5318606 | Myricetin 3-O-beta-D-glucopyranoside | -7.867 | -24.86 |
| 107721 | [Taxiphyllin](https://pubchem.ncbi.nlm.nih.gov/compound/107721) | -7.595 | -36.29 |
| 442793 | [6-Gingerol](https://pubchem.ncbi.nlm.nih.gov/compound/442793) | -7.458 | -41.35 |
| 10095180 | Kaempferol 7-O-glucoside | -7.23 | -39.5 |
| 5280343 | [Quercetin](https://pubchem.ncbi.nlm.nih.gov/compound/5280343) | -6.982 | -41.19 |
| 9064 | [(+)-Catechin](https://pubchem.ncbi.nlm.nih.gov/compound/9064) | -6.915 | -35.15 |
| 8973 | 3-O-Methyl-d-glucose | -6.318 | -34.24 |
| 6438710 | [Sarmentosin](https://pubchem.ncbi.nlm.nih.gov/compound/6438710) | -6.258 | -34.9 |
| 689043 | [Caffeic acid](https://pubchem.ncbi.nlm.nih.gov/compound/689043) | -5.563 | -4.63 |
| 13845942 | [Piperoic acid](https://pubchem.ncbi.nlm.nih.gov/compound/13845942) | -5.503 | -21.68 |
| 287960 | [2-Methyl-3-methylene-1,4-dioxane](https://pubchem.ncbi.nlm.nih.gov/compound/287960) | -5.403 | -23.56 |
| 6453351 | [6-deoxy-D-allose](https://pubchem.ncbi.nlm.nih.gov/compound/6453351) | -5.366 | -31.98 |
| 19309 | [Furaneol](https://pubchem.ncbi.nlm.nih.gov/compound/19309) | -5.364 | -28.6 |
| 72075 | [Bamipine](https://pubchem.ncbi.nlm.nih.gov/compound/72075) | -5.3 | -45.34 |
| 332 | [2-Methoxy-4-vinylphenol](https://pubchem.ncbi.nlm.nih.gov/compound/332) | -5.289 | -32.7 |
| 76323448 | [Dihydropipericide](https://pubchem.ncbi.nlm.nih.gov/compound/76323448) | -5.241 | -45.38 |
| 101878852 | [Cis-Piperettine](https://pubchem.ncbi.nlm.nih.gov/compound/101878852) | -4.949 | -36.28 |
| 451580 | 6-N-Piperidinopurine-9-beta-D-2', 3'-dideoxyribofuranoside | -4.913 | -39.44 |
| 237332 | [5-hydroxymethylfurfural](https://pubchem.ncbi.nlm.nih.gov/compound/237332) | -4.882 | -33.27 |
| 31211 | Zi[ngerone](https://pubchem.ncbi.nlm.nih.gov/compound/31211) | -4.867 | -31.31 |
| 14739 | [Piperamide](https://pubchem.ncbi.nlm.nih.gov/compound/14739) | -4.843 | -44.57 |
| 9796015 | [1-Dehydro-6-gingerdione](https://pubchem.ncbi.nlm.nih.gov/compound/9796015) | -4.789 | -41.73 |
| 9921021 | [Methyl piperate](https://pubchem.ncbi.nlm.nih.gov/compound/9921021) | -4.777 | -33.41 |
| 538757 | 2,4-Dihydroxy-2,5-dimethyl-3(2H)-furanone | -4.75 | -28.46 |
| 119838 | 3,5-dihydroxy-6-methyl-2,3-dihydropyran-4-one | -4.7 | -17.17 |
| 101422868 | [Pipericide](https://pubchem.ncbi.nlm.nih.gov/compound/101422868) | -4.596 | -29.92 |
| 11012859 | [Retrofractamide A](https://pubchem.ncbi.nlm.nih.gov/compound/11012859) | -4.586 | -38.33 |
| 6442405 | [Guineensine](https://pubchem.ncbi.nlm.nih.gov/compound/6442405) | -4.585 | -31.02 |
| 579668 | Methyl 3-(3,4-dihydroxyphenyl)propanoate methyl dihydrocaffeate | -4.564 | -38.41 |
| 273497394 | [Epi-Bisabolol Oxide B](https://pubchem.ncbi.nlm.nih.gov/substance/273497394) | -4.561 | -20.08 |
| 5280443 | [Apigenin](https://pubchem.ncbi.nlm.nih.gov/compound/5280443) | -4.547 | -34.95 |
| 14162526 | Brachyamide B | -4.546 | -14.95 |
| 9974595 | [Pipernonaline](https://pubchem.ncbi.nlm.nih.gov/compound/9974595) | -4.512 | -33.37 |
| 5320621 | [Piperlonguminine](https://pubchem.ncbi.nlm.nih.gov/compound/5320621) | -4.379 | -38.12 |
| 7362 | [Furfural](https://pubchem.ncbi.nlm.nih.gov/compound/7362) | -4.363 | -24.02 |
| 21648 | [4-(4-Hydroxyphenyl)-2-butanone](https://pubchem.ncbi.nlm.nih.gov/compound/21648) | -4.356 | -32.54 |
| 5282110 | [Cinnamyl acetate](https://pubchem.ncbi.nlm.nih.gov/compound/5282110) | -4.289 | -26.92 |
| 94378 | [Paradol](https://pubchem.ncbi.nlm.nih.gov/compound/94378) | -4.19 | -42.7 |
| 222284 | [Beta-sitosterol](https://pubchem.ncbi.nlm.nih.gov/compound/222284) | -4.127 | -36.48 |
| 252448317 | [(R)-4-Hydroxy-Methyl-Benzenepropanol](https://pubchem.ncbi.nlm.nih.gov/substance/252448317) | -4.076 | -31.75 |
| 10466747 | 7-epi-eudesm-4(15)-ene-1beta,6beta-diol | -4.036 | -26.76 |
| 21580213 | [Piperolein B](https://pubchem.ncbi.nlm.nih.gov/compound/21580213) | -4.015 | -40.62 |
| 636537 | [Piperyline](https://pubchem.ncbi.nlm.nih.gov/compound/636537) | -3.96 | -31.32 |
| 442985 | [Solasodine](https://pubchem.ncbi.nlm.nih.gov/compound/442985) | -3.92 | -30.04 |
| 2750488 | [2-Propenoic acid](https://pubchem.ncbi.nlm.nih.gov/compound/2750488) | -3.884 | -22.3 |
| 637858 | [Piperlongumine](https://pubchem.ncbi.nlm.nih.gov/compound/637858) | -3.869 | -18.92 |
| 6432404 | [(+)-gamma-cadinene](https://pubchem.ncbi.nlm.nih.gov/compound/6432404) | -3.858 | -16.71 |
| 6428423 | [Cadinol](https://pubchem.ncbi.nlm.nih.gov/compound/6428423) | -3.835 | -18.44 |
| 3084311 | [Delta-Cadinol](https://pubchem.ncbi.nlm.nih.gov/compound/3084311) | -3.835 | -18.44 |
| 5284507 | [NEROLIDOL](https://pubchem.ncbi.nlm.nih.gov/compound/5284507) | -3.799 | -38.16 |
| 5371378 | 4-hydroxy-3,5,6-trimethyl-4-[(*E*)-3-oxobut-1-enyl]cyclohex-2-en-1-one | -3.754 | -38.66 |
| 643820 | [Nerol](https://pubchem.ncbi.nlm.nih.gov/compound/643820) | -3.751 | -31.07 |
| 442360 | [L-alpha-Curcumene](https://pubchem.ncbi.nlm.nih.gov/compound/442360) | -3.676 | -26.41 |
| 3084581 | (2-methoxy-4-propylphenyl) acetate | -3.675 | -29.67 |
| 92812 | [Ledol](https://pubchem.ncbi.nlm.nih.gov/compound/92812) | -3.601 | -17.52 |
| 5375928 | (*E*)-1-(3-methoxy-4-trimethylsilyloxyphenyl)dec-4-en-3-one | -3.584 | -34.69 |
| 17100 | [Alpha-terpineol](https://pubchem.ncbi.nlm.nih.gov/compound/17100) | -3.554 | -21.65 |
| 12078508 | Hexanoic acid | -3.54 | -30.21 |
| 61586 | 4-(ethoxymethyl)-2-methoxyphenol | -3.534 | -22.22 |
| 17429 | [N-Formylpiperidine](https://pubchem.ncbi.nlm.nih.gov/compound/17429) | -3.487 | -17.99 |
| 6429302 | [Trans-alpha-Bergamotene](https://pubchem.ncbi.nlm.nih.gov/compound/6429302) | -3.47 | -26.96 |
| 21580214 | (*E*)-9-(1,3-benzodioxol-5-yl)-1-pyrrolidin-1-ylnon-8-en-1-one | -3.438 | -33.39 |
| 111037 | [Terpinyl acetate](https://pubchem.ncbi.nlm.nih.gov/compound/111037) | -3.42 | -20.18 |
| 605629 | Methyl 2-(4-*tert*-butylphenyl)acetate | -3.406 | -30.14 |
| 12315492 | [Beta-Sesquiphellandrene](https://pubchem.ncbi.nlm.nih.gov/compound/12315492) | -3.358 | -21.69 |
| 9855795 | [Valencene](https://pubchem.ncbi.nlm.nih.gov/compound/9855795) | -3.341 | -23.12 |
| 12306054 | [Delta-Amorphene](https://pubchem.ncbi.nlm.nih.gov/compound/12306054) | -3.233 | -17 |
| 6549 | [Linalool](https://pubchem.ncbi.nlm.nih.gov/compound/6549) | -3.186 | -27.15 |
| 19725 | [Copaene](https://pubchem.ncbi.nlm.nih.gov/compound/19725) | -3.154 | -22.48 |
| 5470187 | [Zerumbone](https://pubchem.ncbi.nlm.nih.gov/compound/5470187) | -3.089 | -16.53 |
| 91723653 | [Isogermacrene D](https://pubchem.ncbi.nlm.nih.gov/compound/91723653) | -3.01 | -15.58 |
| 18818 | [SABINENE](https://pubchem.ncbi.nlm.nih.gov/compound/18818) | -3.002 | -11.59 |
| 10910653 | [Ledene](https://pubchem.ncbi.nlm.nih.gov/compound/10910653) | -2.941 | -19.33 |
| 1742210 | [Caryophyllene oxide](https://pubchem.ncbi.nlm.nih.gov/compound/1742210) | -2.886 | -17.4 |
| 5280489 | [Beta-carotene](https://pubchem.ncbi.nlm.nih.gov/compound/5280489) | -2.885 | -20.44 |
| 91354 | [Aromadendrene](https://pubchem.ncbi.nlm.nih.gov/compound/91354) | -2.869 | -14.21 |
| 92776 | [Zingiberene](https://pubchem.ncbi.nlm.nih.gov/compound/92776) | -2.832 | -31.86 |
| 5281520 | [Humulene](https://pubchem.ncbi.nlm.nih.gov/compound/5281520) | -2.831 | -21.09 |
| 12306048 | [Alpha-Cadinene](https://pubchem.ncbi.nlm.nih.gov/compound/12306048) | -2.821 | -25.31 |
| 985 | [Palmitic acid](https://pubchem.ncbi.nlm.nih.gov/compound/985) | -2.772 | -22.21 |
| 6432744 | [Beta.-Sitosterol](https://pubchem.ncbi.nlm.nih.gov/compound/6432744) | -2.744 | -28.51 |
| 99474 | [Diosgenin](https://pubchem.ncbi.nlm.nih.gov/compound/99474) | -2.694 | -25.15 |
| 91753138 | [(Z)-Calacorene](https://pubchem.ncbi.nlm.nih.gov/compound/91753138) | -2.667 | -21.49 |
| 31289 | [NONANAL](https://pubchem.ncbi.nlm.nih.gov/compound/31289) | -2.599 | -32.42 |
| 5366244 | [Phytol](https://pubchem.ncbi.nlm.nih.gov/compound/5366244) | -2.508 | -41.72 |
| 21155918 | [Carpesterol](https://pubchem.ncbi.nlm.nih.gov/compound/21155918) | -2.499 | -43.99 |
| 10104370 | [Beta-Bisabolene](https://pubchem.ncbi.nlm.nih.gov/compound/10104370) | -2.449 | -26.45 |
| 12306049 | [(+)-alpha-muurolene](https://pubchem.ncbi.nlm.nih.gov/compound/12306049) | -2.331 | -22.27 |
| 2682 | [1-Hexadecanol](https://pubchem.ncbi.nlm.nih.gov/compound/2682) | -2.324 | -34.86 |
| 6654 | [Alpha-pinene](https://pubchem.ncbi.nlm.nih.gov/compound/6654) | -2.278 | -11.71 |
| 2758 | [Eucalyptol](https://pubchem.ncbi.nlm.nih.gov/compound/2758) | -1.797 | -8.66 |
| 5365570 | (6*Z*,9*Z*)-pentadeca-6,9-dien-1-ol | -1.759 | -38.98 |
| 5281516 | [Alpha-Farnesene](https://pubchem.ncbi.nlm.nih.gov/compound/5281516) | -1.573 | -24.88 |
| 8209 | [1-Tetradecanol](https://pubchem.ncbi.nlm.nih.gov/compound/8209) | -1.373 | -29.7 |
| 5281243 | [Lutein](https://pubchem.ncbi.nlm.nih.gov/compound/5281243) | -1.105 | -27.39 |
| 5280794 | [Stigmasterol](https://pubchem.ncbi.nlm.nih.gov/compound/5280794) | -0.972 | -24.98 |
| 11141599 | [(E)-Piperolein A](https://pubchem.ncbi.nlm.nih.gov/compound/11141599) | -0.81 | -33.29 |
